# Prognostic value of lymphocyte-to-monocyte ratio in acute ischemic stroke: a systematic review and meta-analysis

**DOI:** 10.3389/fneur.2025.1567112

**Published:** 2025-05-07

**Authors:** Chengli Tian, Yilin Yang, Jun Wan, Ran Wang, Ke Zhou, Yuanli Li, Wenlong Guo, Huijun Li, Yu Zhang

**Affiliations:** ^1^Center for Evidence-Based Medicine, Affiliated Hospital of Chengdu University, Chengdu, Sichuan, China; ^2^Department of Critical Care Medicine, Affiliated Hospital of Chengdu University, Chengdu, Sichuan, China

**Keywords:** lymphocyte-to-monocyte, LMR, acute ischemic stroke, AIS, poor functional outcome, stroke, prognosis

## Abstract

**Background:**

The findings from studies exploring the prognostic relevance of the lymphocyte-to-monocyte ratio (LMR) in individuals with acute ischemic stroke (AIS) have shown variability. We aimed to conduct a meta-analysis to determine the prognostic significance of LMR in this patient population.

**Methods:**

We carried out a meta-analysis utilizing information from major databases, including PubMed, Embase, and Web of Science until October 26, 2024. Effect sizes, represented as odds ratios (ORs) along with their corresponding 95% confidence intervals (CI), were synthesized employing a random-effects model in Review Manager Version 5.4. To investigate possible sources of variability, we conducted subgroup analyses. Additionally, publication bias was assessed through the use of a funnel plot. Poor functional outcome at 3 months, as indicated via a modified Rankin Scale score of ≥3, was the main outcome. A moderate to severe stroke, determined by a National Institutes of Health Stroke Scale score of ≥6, was the secondary outcome.

**Results:**

Six trials totaling 1,225 individuals were included in our analysis. In AIS patients, we discovered a significant correlation between lower LMR and poorer functional outcome at 3 months, with an OR of 0.63, 95% CI of 0.49 to 0.80, and a *p*-value of 0.0002. Additionally, lower LMR may be associated with developing moderate to severe stroke, with an OR of 0.89 (95% CI: 0.82–0.97; *p* = 0.008). In subgroup analyses with an LMR cutoff, a significant association was observed between lower LMR and greater functional impairment in AIS patients, with an odds ratio of 0.74 (95% CI: 0.62–0.88; *p* = 0.0005) for LMR ≥ 3 and 0.54 (95% CI: 0.47–0.61; *p* < 0.00001) for LMR < 3. Additionally, when country-stratified, Asian continued to have a significant correlation between worse functional outcome and lower LMR (OR 0.62, 95% CI: 0.50–0.77, *p* < 0.0001).

**Conclusion:**

This meta-analysis indicated that LMR was a prognostic factor for clinical outcomes in AIS patients.

## Introduction

A stroke is characterized by its abrupt onset, representing a sudden neurological disruption resulting from the abrupt cessation of cerebral blood flow, which leads to a wide spectrum of neurological deficits (1). Strokes are generally classified into two main categories: ischemic and hemorrhagic. Of these, ischemic strokes account for approximately 80% of all cases, with an alarmingly high global incidence of over 13.7 million cases annually, leading to around 5.5 million deaths each year (2). Although strokes can affect individuals across all age groups, they primarily affect older adults. However, there has been a concerning rise in the incidence among younger populations (2, 3). Among the different types of stroke, acute ischemic stroke (AIS) is the most common, placing a significant strain on healthcare systems due to its high morbidity and mortality rates, as well as the considerable risks of fatal outcomes and long-term disabling effects (4). Thus, identifying reliable prognostic markers is crucial for informed clinical decision-making and personalized patient care.

Increasing evidence indicates that AIS triggers an inflammatory response (5–7). There is growing interest in the part inflammatory indicators play in the pathophysiology and outcome of AIS. A cost-effective and easily accessible composite indicator of inflammation in cerebrovascular disease, the lymphocyte-to-monocyte ratio (LMR), which was computed by dividing the lymphocyte count by the monocyte count, integrates the prognostic significance of individual lymphocyte and monocyte levels in relation to AIS (8). Previous research has indicated an association between LMR and the severity as well as outcomes of inflammation-related maladies such as myocardial infarction, chronic autoimmune diseases, and peripheral ischemia (9–11).

The link between LMR and adverse outcomes in AIS patients continues to be a subject of debate. Although a number of studies have shown a correlation between AIS patients’ inferior prognosis and lower LMR levels (12–15), others have failed to establish a significant link between LMR and negative clinical outcomes (16). In light of these discrepant results, we carried out this meta-analysis to delve deeper into the relationship between LMR and adverse outcomes among AIS patients.

## Methods

In accordance with the PRISMA statement, this systematic review and meta-analysis has been registered in the PROSPERO database under the registration number CRD42025637708 (17).

### Data sources and search strategy

A thorough systematic review was performed utilizing various databases, such as PubMed, Web of Science, and Embase. Additionally, we hand-searched the bibliographies of all selected studies to locate further pertinent articles. Literature from the inception of these databases to October 26, 2024, was included in the search. The full search strategies are presented in Supplementary Table S1.

### Inclusion and exclusion criteria

The selection process for studies was influenced by these criteria: (1) Patients must be clinically confirmed to have ischemic stroke; (2) The LMR was assessed either upon admission or during the hospital stay for ischemic stroke; (3) Outcome measures included poor functional outcome, or moderate to severe stroke; (4) Participants had to be adults aged 18 years or older. Any studies that met the following criteria were left out of the meta-analysis: (1) They exclusively included patients with hemorrhagic stroke; (2) They were conference abstracts; (3) They were experimental or interventional studies, review articles, preprint studies, or case reports; (4) They lacked sufficient data; (5) They involved non-human subjects.

### Outcomes

The main result at 3 months was poor functional outcome, which was characterized as a Modified Rankin Scale (MRS) score of three or above. A stroke score of 6 or higher on the National Institutes of Health Stroke Scale (NIHSS) was considered moderate to severe, and this was the secondary outcome.

### Data extraction

Data extraction was done separately by two researchers, RW and CT, from the chosen studies. They collected specific details, including the lead author’s name, publication date, verification methodology, mean participant age, geographical location of the study, total number of participants, gender breakdown, measurement outcomes, the LMR Youden Index, LMR threshold levels, Area Under the Curve (AUC), odds ratio (OR) for adverse functional outcome and moderate to severe stroke cases, and their associated 95% confidence interval (CI). When studies provided both univariate and multivariate regression analyses, the latter was utilized to derive the cumulative OR. To ensure data accuracy, a third researcher (YL) conducted an independent verification. Within the collaboration, disagreements between the original reviewers were settled via cooperative discussion.

### Assessment of quality

Cohort study quality was rated by two reviewers (RW and CT) adopting the Newcastle–Ottawa Quality Assessment Scale (NOS), with scores ranging from 0 to 9. Studies scoring 7 or higher were deemed high quality (18). Each study was independently evaluated by the reviewers in line with the designated scale. Any differences were reconciled by means of discussions with an additional author (YL).

### Statistical analysis

RevMan 5.4.1 from The Cochrane Collaboration was used for statistical analysis. For categorical data, the OR and associating 95% CI were determined. The degree of variability was quantified by using the I^2^ statistic to calculate the percentage of variability attributable to study variability, with a *p*-value of less than 0.05 being deemed statistically significant. Because of the diversity shown in the trials, a random-effects model was used to synthesize the findings. To determine the degree of heterogeneity, I^2^ statistics and Chi-square tests were applied, with a study considered significantly heterogeneous when *p* < 0.05 and I^2^ exceeded 50%. Forest plots were created to visualize the estimated effect sizes along with their 95% CI. By employing these rigorous statistical techniques, our goal was to deliver a thorough and comprehensive analysis of the data. We visually inspected the funnel plot to qualitatively assess the potential effects of small studies. Additionally, we performed a sensitivity analysis by removing studies one at a time in order to examine how each study affected the overall impact size. Moreover, the egger test (19) was applied for quantitative analysis, facilitating a meticulous assessment of any bias that might arise from small-study effects.

### Subgroup analysis

We conducted a subgroup analysis of the primary outcome according to age, country, LMR cutoff values, and the specific characteristics of the study subjects.

## Results

### Study inclusion

**Figure 1:**
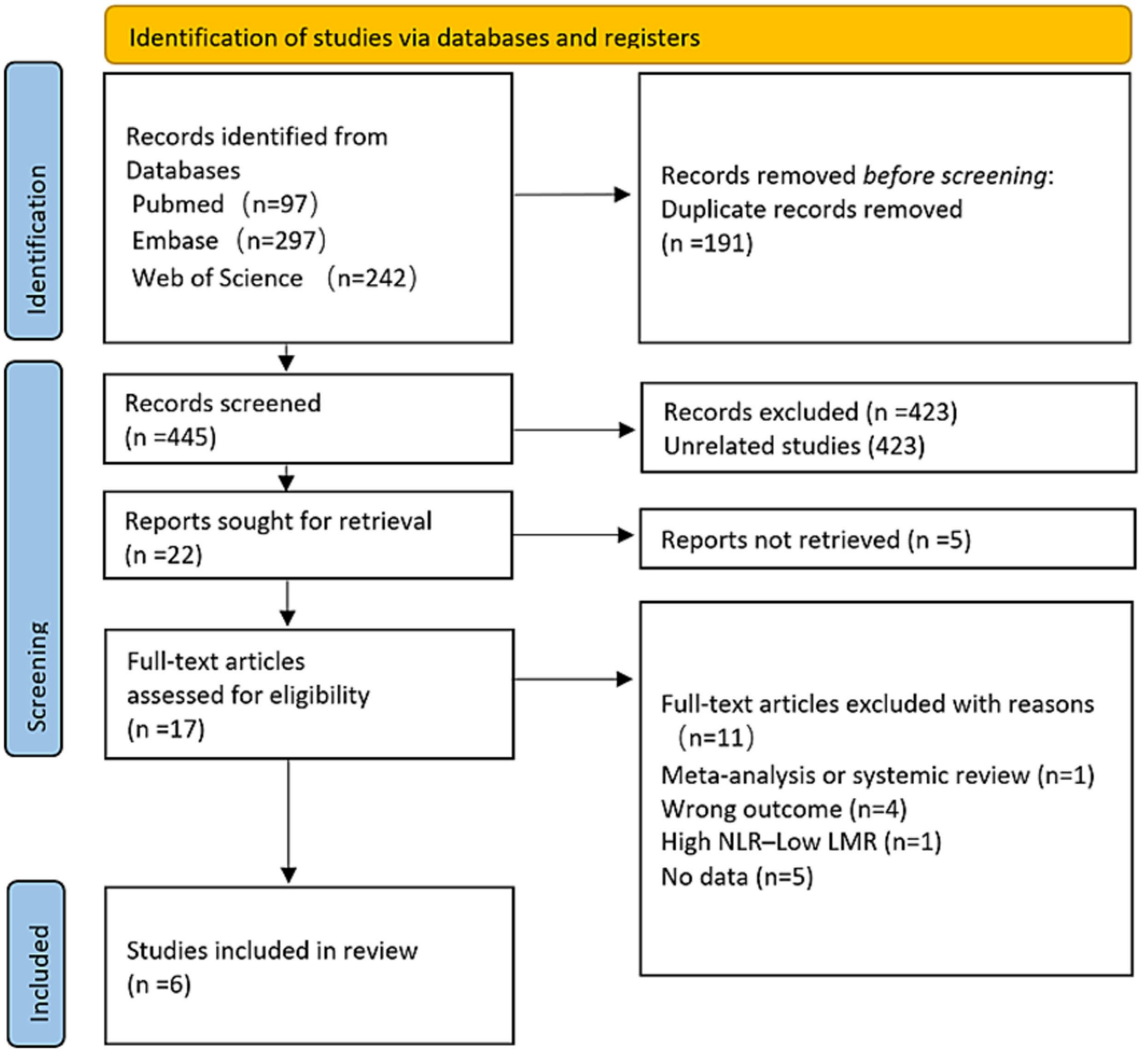
PRISMA study flow diagram.

Figure 1 illustrates the flowchart detailing the research design. A comprehensive literature search, employing specific keywords, initially identified 636 articles. After eliminating duplicates, 191 studies were excluded. Subsequently, 423 irrelevant articles were excluded. Among the remaining 22, 5 were excluded for inaccessible full-texts. Following a thorough review, 11 additional articles were disqualified for not fulfilling the predetermined inclusion requirements. In the end, the meta-analysis had six publications (12–15, 20, 21).

### Summary of studies

Table 1 offers a comprehensive summary of details of the included research. In total, 1,225 individuals diagnosed with AIS were documented across six retrospective studies. All included studies adjusted for potential confounders through multivariate models. The initial studies predominantly focused on populations from China, the UK (21), and Trnava, Slovakia (12). The primary source of data was the Department of Neurology at Chengde Medical University’s affiliated hospital (14, 15). The analysis revealed a higher prevalence of male patients with AIS, with the mean age exceeding 50 years. Stroke diagnoses were confirmed through clinical evaluations, imaging studies, and comprehensive diagnostic assessments.

**Table 1:** Basic statistical characteristics of included studies.

Studies	Country	Age, year	*N* (F/M)	Confirmed method	AUC	cutoff value	Youden index	Outcome measures	NOS
Guan et al., 2023 (13)	China	71.0 (63.0–81.0)	101 (37/64)	-	0.789	1.92	0.531	MRS	9
Ren et al., 2017 (14)	China	61.0 (55.0–74.0)	512 (198/314)	MRI	0.826	2.99	0.559	MRS NIHSS	7
Lux et al., 2020 (21)	UK	66.4 (49.7–83.1)	121 (58/63)	-	-	2.00	0.300	MRS	6
Pinčáková et al., 2022 (12)	Trnava, Slovakia	72.0 (69.0–81.0)	179 (90/89)	CTA MRA DSA	0.616	3.27	0.280	MRS	7
Ren et al., 2017 (15)	China	56.0 (52.0–69.0)	108 (32/76)	MRI CT	0.767	3.48	0.521	MRS NIHSS	9
Zhang et al., 2024 (20)	China	69.1 (56.8–81.4)	204 (73/131)	-	-	-	-	MRS	8

CT, computed tomography; MRI, magnetic resonance imaging; N (F/M), number of patients (female/male); AUC, area under the curve; CTA, computed tomography angiography; MRA, magnetic resonance angiography; DSA, digital subtraction angiography; MRS, modified Rankin Scale; NIHSS, the National Institute of Health Stroke Scale; NOS, Newcastle-Ottawa Scale.

### Overall assessment of evidence quality

The evaluation of the cohort investigations employing the NOS This led to scores of 9 in two studies (13, 20), 8 in one study (12), 7 in two studies (14, 15), and 6 in another one (21). The differences in scores primarily depended on the extent to which confounders were controlled and the rigor of participant follow-up (Supplementary Table S2).

### Overall efficacy

**Figure 2:**
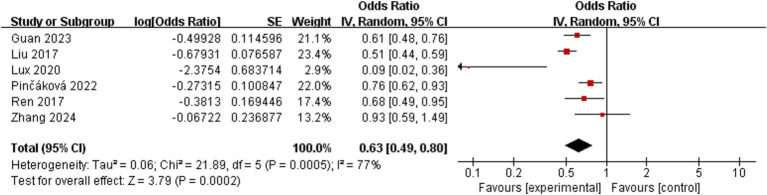
Forest plot of the association between lymphocyte-to-monocyte ratio and poor functional outcome at 3 months.

The incidence of poor functional outcome in AIS patients at 3 months was the main outcome that was measured. This was determined by combining effect sizes from six studies, which collectively included 1,225 participants. The pooled OR for these studies was 0.63 (95% CI: 0.49–0.80, *p* = 0.0002), as shown in Figure 2. According to this finding, AIS patients who have a lower LMR are more likely to experience poorer functional results at 3 months. Notably, there was considerable variation among the trials (I^2^ = 77%, *p* = 0.0005).

**Figure 3:**
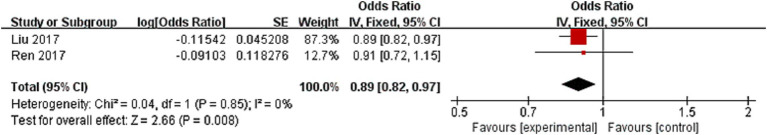
Forest plot of the association between lymphocyte-to-monocyte ratio and moderate to severe stroke.

The secondary outcome was the occurrence of moderate to severe stroke in patients with AIS. Two studies evaluated moderate to severe stroke as an outcome, resulting in a pooled OR of 0.89 (95% CI: 0.82–0.97, *p* = 0.008), according to Figure 3. There was no difference between the investigations (I^2^ = 0, *p* = 0.85).

### Subgroup analyses

We performed a subgroup analysis focusing on studies that used poor functional outcome at 3 months as the primary outcome measure. The results, shown in Supplementary Figure S1, reveal that the aggregated OR for poor functional outcome was 0.62 (95% CI: 0.50–0.77; *p* < 0.0001) for studies conducted in Asian, and 0.30 (95% CI: 0.04–2.30; *p* = 0.24) for studies conducted in non-Asian. Among all included studies, two reported a LMR cutoff value ≥ 3, yielding a pooled OR for poor functional outcome of 0.74 (95% CI: 0.62–0.88; *p* = 0.0005). In contrast, the LMR cutoff values reported in the other three studies ranged between 0 and 3, resulting in a combined OR of 0.50 (95% CI: 0.35–0.70; *p* < 0.0001). According to sensitivity analysis, the Lux et al. (21) study affected the overall findings, most likely as a function of the timing of the LMR measurements. In their study, LMR was measured 24 h after mechanical thrombectomy (MT), whereas other included studies measured LMR at admission or during the initial hospital stay (13, 14). This variability in timing of LMR measurement may have contributed to the observed heterogeneity. Excluding this study resulted in a pooled OR of 0.54 (95% CI: 0.47–0.61, *p* < 0.00001), based on Supplementary Figure S2. One study did not specify an LMR cutoff value. Furthermore, a subgroup analysis based on age (Supplementary Figure S3) demonstrated that the pooled OR for poor functional outcome at 3 months was 0.65 (95% CI: 0.46–0.93; *p* = 0.02) for patients aged 65 and older, and 0.57 (95% CI: 0.43–0.75; *p* < 0.0001) for those younger than 65. The analysis also included two studies focusing on AIS patients who underwent MT, resulting in an OR of 0.30 (95% CI: 0.04–2.30; *p* = 0.24). Additionally, two trials including AIS patients evaluated the impact of recombinant tissue plasminogen activator (rt-PA), resulting in an OR of 0.77 (95% CI: 0.57–1.03; *p* = 0.08). Another study focused on AIS patients who received endovascular therapy (EVT), yielding a pooled OR of 0.61 (95% CI: 0.48–0.76). Lastly, one study addressed AIS patients more generally, reporting a pooled OR of 0.51 (95% CI: 0.44–0.59), as illustrated in Supplementary Figure S4. All pooled results are summarized in Table 2.

**Table 2:** Subgroup analysis results of poor functional outcome at 3 months.

Groups	*N*	Model	Pooled OR (95%CI)	*p*	Heterogeneity (P, I2)	Conclusion
Total	6	Random	0.63 (0.49,0.80)	0.0002	0.0005,77%	Positive
Asian	4	Random	0.62 (0.50, 0.77)	*p* < 0.0001	0.04,63%	Positive
No-Asian	2	Random	0.30 (0.04, 2.30)	0.24	0.002,89%	Negative
Age ≥ 65	4	Random	0.65 (0.46, 0.93)	0.02	0.006,76%	Positive
Age < 65	2	Random	0.57 (0.43, 0.75)	*p* < 0.0001	0.11,61%	Positive
LMR ≥ 3	2	Fixed	0.74 (0.62, 0.88)	0.0005	0.58,0%	Positive
LMR < 3	3	Random	0.50 (0.35, 0.70)	*p* < 0.0001	0.02,76%	Positive
LMR < 3, Lux 2020 trials excluded	2	Fixed	0.54 (0.47, 0.61)	*p* < 0.00001	0.19,41%	Positive
AIS patients after MT	2	Random	0.30 (0.04, 2.30)	0.24	0.002,89%	Negative
AIS patients after rt-PA	2	Fixed	0.76 (0.58, 1.00)	0.05	0.28,14%	Negative
AIS patients after EVT	1	Random	0.61 (0.48, 0.76)	*p* < 0.0001	-	Positive
AIS patients	1	Random	0.51 (0.44, 0.59)	*p* < 0.00001	-	Positive

*N, number; CI, confidence interval; OR, odds ratio; LMR, lymphocyte-to-monocyte ratio; MT, mechanical thrombectomy; rt-PA, recombinant tissue plasminogen activator; EVT, endovascular treatment.*

### Sensitivity analysis and publication bias

One study at a time was eliminated in order to do a sensitivity analysis. The findings of this analysis are presented in Supplementary Table S3. The sensitivity analysis indicated that the pooled OR for studies reporting poor functional outcome remained stable, with no significant changes observed after the exclusion of any individual study. Additionally, publication bias was evaluated and is presented in Supplementary Figures S5, S6. No substantial bias was identified in the meta-analysis.

## Discussion

To our knowledge, this meta-analysis represents the pioneering investigation in its field that, by implementing rigorous search protocols, established the absence of prior systematic reviews—even those tangentially examining inflammatory biomarkers in acute ischemic stroke, including information from six distinct investigations. Our findings suggest that a lower LMR is associated with worse functional outcome at 3 months and may also indicate a higher likelihood of moderate to severe stroke occurrence.

The relationship between the LMR and AIS has not yet been the subject of any meta-analysis. Our study uniquely focuses on this relationship within the AIS population, proving that LMR may indicate a useful predictor of poor functional outcome in patients with AIS. Furthermore, our findings indicate that reduced LMR levels are associated with a higher risk of progressing to moderate to severe stroke. Additionally, the place of origin, research parameters, and cutoff values were among the factors that affected the association between LMR and AIS, according to our analysis.

The LMR is a marker that reflects the proportion of lymphocytes relative to monocytes in the peripheral circulation. This ratio serves as an inflammatory biomarker and has been associated with inflammation, disease activity, and prognosis in various clinical conditions (22). In different clinical settings, variations in LMR can aid physicians in assessing a patient’s immune response. For example, in patients with cancer, higher LMR levels are often associated with more favorable prognosis, as elevated LMR may indicate a more robust immune response (23). On the other hand, a decreased LMR may signify heightened inflammation or immune suppression, factors commonly associated with worse treatment outcomes and lower survival rates. Additionally, LMR has been used to assess the risk of cardiovascular diseases, and evaluate the activity of other chronic inflammatory conditions (22, 24, 25). Clinical observation indicated that lymphocyte levels demonstrated neuroprotective capacities, which may facilitate the restoration of compromised neural functions (26). In contrast, monocytes, recognized as critical immune modulators that amplify post-stroke inflammatory cascades, demonstrate the capacity to migrate into ischemic regions and exacerbate cerebral injury (27). Clinical investigations have further established that elevated monocyte concentrations correlate with unfavorable therapeutic outcomes in acute ischemic stroke, as supported by a study (28). Consequently, decreased LMR levels may serve as a valuable prognostic indicator for AIS outcomes. Clinicians can gain important information about a patient’s general health and the course of their sickness by tracking blood indicators like LMR.

Our analysis suggests that the LMR is a valuable prognostic marker for predicting poor functional outcome 3 months following AIS. It is crucial to understand that a variety of factors, such as comprehensive rehabilitation efforts, the initial severity of the stroke, and the specific emergency interventions administered, affect these results (29–34). Additionally, several potential confounding factors warrant consideration. For instance, stroke subtype may differentially influence inflammatory responses and subsequent LMR dynamics (35). Baseline comorbidities such as diabetes, hypertension, or chronic inflammatory conditions may modulate both lymphocyte and monocyte counts, thereby confounding the LMR-outcome association (36, 37). Pre-stroke functional status, as assessed by metrics like the MRS prior to the index event, might independently predict recovery trajectories and interact with LMR’s prognostic utility (38). Furthermore, variations in acute therapies may alter the inflammatory milieu and LMR values, necessitating careful adjustment in future studies (39, 40). Our findings indicate that a lower LMR cutoff value is more predictive of poorer functional outcome at 3 months in AIS patients. Consistent with this, Guan et al. (13) showed that a threshold value of 1.92 was found for the independent correlation between a lower LMR and a higher likelihood of poor functional outcome 3 months after a stroke. Similarly, Lux et al. (21) reported that a lower LMR cutoff of 2, with a sensitivity of 80% and specificity of 50%, was also associated with AIS patients having a higher chance of experiencing poor functional outcome at 3 months.

We conducted the subgroup analyses to identify and minimize factors contributing to heterogeneity. In the Asian group, a lower LMR was associated with worse functional outcomes, whereas this relationship was non-significant in the non-Asian group, potentially due to the smaller sample size and increased risk of Type II error. Biological factors such as genetic differences in inflammation and platelet biology, as well as healthcare system and environmental variations, may also influence these findings. Furthermore, testing of different LMR cutoff values revealed that a cutoff of 3 was most predictive of disease progression and prognosis, with lower cutoff values showing stronger associations. LMR demonstrated greater prognostic utility in younger patients, likely due to preserved immune function, while aging, comorbidities, and polypharmacy in the elderly may diminish its predictive value. Through the subgroup analyses, we examined potential sources of heterogeneity and identified factors such as country of origin, age and LMR cutoff value as plausible contributors.

Despite our adherence to rigorous standards in study selection, data extraction, and quality assessment, several limitations were inherent in our study. A key limitation of this meta-analysis is the retrospective design of most included studies, which may introduce selection bias and confounding factors, potentially affecting the reliability of the pooled effect estimates. First, subgroup analyses, such as those based on ethnicity and LMR cutoff values, should be interpreted with caution due to limited study numbers, particularly in the non-Asian subgroup, which had only two studies, limiting statistical power. Second, variations in LMR cutoff values and measurement timing across studies contribute to heterogeneity, underscoring the need for standardized protocols in future research to improve comparability and reduce variability. Moreover, as most of the studies in this analysis were based in Asia, particularly China, caution was needed when generalizing these findings. Additionally, the random-effects model highlighted significant variability across studies, suggesting potential publication bias.

The use of the LMR as a biomarker in AIS patients offers significant clinical advantages. LMR can help identify patients at higher risk for moderate to severe stroke and poor functional outcomes, enabling personalized management strategies. It also aids in treatment decisions, guiding clinicians to consider more aggressive interventions for poor prognostic cases. Integration of LMR with point-of-care testing devices, particularly in emergency settings, facilitates real-time risk assessment and decision-making during the critical golden hour. Additionally, educating patients on the implications of LMR can improve patient engagement and treatment adherence, ultimately enhancing outcomes and recovery.

## Conclusion

Three months after an acute ischemic stroke, our study unequivocally showed a substantial correlation between the LMR and poor functional outcome. Specifically, lower LMR was associated with poorer functional outcome and a higher risk of developing moderate to severe stroke. These findings indicate that LMR may be a valuable prognostic tool for guiding management and identifying high-risk acute ischemic stroke patients who require closer post-treatment monitoring.
