# Supplementary material for: Prognostic value of lymphocyte-to-monocyte ratio in acute ischemic stroke: a systematic review and meta-analysis
Source: Front Neurol. 2025 May 7;16:1567112. doi: 10.3389/fneur.2025.1567112 (PMC12092227; doi:10.3389/fneur.2025.1567112)
Supplement: Supplementary file 2 [file Table_2.docx]

Supplementary Material

| Table S1: Search strategy. |
| --- |
| Table S2: Sensitivity analysis of meta-analysis between LMR and poor functional outcome at 3 months. |
| Table S3: The Newcastle-Ottawa Quality Assessment Scale score for cohort studies. |
| Figure S1. Forest plot of Asian and non‐Asian populations subgroup analysis. |
| Figure S2. Forest plot of the best cutoff value subgroup analysis. |
| Figure S3. Forest plot of age subgroup analysis. |
| Figure S4. Forest plot of characteristics of the research object subgroup analysis. |
| Figure S5: Funnel plot of the association between Lymphocyte-to-Macrophage ratio and poor functional outcome at 3 months.  Figure S6: Funnel plot of the association between Lymphocyte-to-Macrophage ratio and moderate to severe stroke. |

**Table S1: Search strategy.**

| **PubMed** | | |
| --- | --- | --- |
|  | "Lymphocytes"[Mesh] | 589073 |
|  | Lymphocyte [Title/Abstract] OR Lymphoid Cell [Title/Abstract] OR Cell, Lymphoid [Title/Abstract] | 194419 |
|  | #1 OR #2 | 674557 |
|  | "Monocytes"[Mesh] | 70181 |
|  | Monocyte [Title/Abstract] | 78456 |
|  | #4 OR #5 | 120179 |
|  | #3 AND #6 | 29094 |
|  | L/M ratio [Title/Abstract] OR lymphocyte to monocyte ratio [Title/Abstract] OR lymphocyte monocyte ratio [Title/Abstract] OR LMR[Title/Abstract] | 2640 |
|  | #7 OR #8 | 29969 |
|  | "Ischemic Stroke"[Mesh] | 14953 |
|  | Ischemic Strokes[Title/Abstract] OR Stroke, Ischemic[Title/Abstract] OR Ischaemic Stroke[Title/Abstract] OR Ischaemic Strokes[Title/Abstract] OR Stroke, Ischaemic[Title/Abstract] OR Acute Ischemic Stroke[Title/Abstract] OR Acute Ischemic Strokes[Title/Abstract] OR Ischemic Stroke, Acute[Title/Abstract] OR Stroke, Acute Ischemic[Title/Abstract] OR Cerebral Stroke[Title/Abstract] OR Cerebral Strokes[Title/Abstract] OR Stroke, Cerebral[Title/Abstract] OR Strokes, Cerebral[Title/Abstract] OR Stroke, Acute[Title/Abstract] OR Acute Stroke[Title/Abstract] OR Acute Strokes[Title/Abstract] OR Strokes, Acute[Title/Abstract] OR Cerebrovascular Accident, Acute[Title/Abstract] OR Acute Cerebrovascular Accident[Title/Abstract] OR Acute Cerebrovascular Accidents[Title/Abstract] OR Cerebrovascular Accidents, Acute[Title/Abstract] | 56006 |
|  | #10 OR #11 | 63079 |
|  | #9 AND #12 | 97 |
| **EMBASE** | | |
|  | 'lymphocyte'/exp | 1118430 |
|  | 'lymphocyte':ab,ti OR 'lymphoid cell':ab,ti OR 'cell, lymphoid':ab,ti | 254394 |
|  | #1 OR #2 | 1224825 |
|  | 'monocyte'/exp | 148433 |
|  | 'monocyte':ab,ti | 105144 |
|  | #4 OR #5 | 195344 |
|  | #3 AND #6 | 73459 |
|  | 'lymphocyte monocyte ratio'/exp | 2548 |
|  | 'lymphocyte to monocyte ratio':ab,ti OR 'lymphocyte monocyte ratio':ab,ti OR 'lmr':ab,ti | 3378 |
|  | #8 OR #9 | 3981 |
|  | #7 OR #10 | 74814 |
|  | 'ischemic stroke'/exp | 36857 |
|  | 'ischemic strokes':ab,ti OR 'stroke, ischemic':ab,ti OR 'ischaemic stroke':ab,ti OR 'ischaemic strokes':ab,ti OR 'stroke, ischaemic':ab,ti OR 'acute ischemic stroke':ab,ti OR 'acute ischemic strokes':ab,ti OR 'ischemic stroke, acute':ab,ti OR 'stroke, acute ischemic':ti OR 'cerebral stroke':ti,ab OR 'cerebral strokes':ab,ti OR 'stroke, cerebral':ab,ti OR 'strokes, cerebral':ab,ti OR 'stroke, acute':ab,ti OR 'acute stroke':ab,ti OR 'acute strokes':ab,ti OR 'strokes, acute':ab,ti OR 'cerebrovascular accident, acute':ab OR 'acute cerebrovascular accident':ab,ti OR 'acute cerebrovascular accidents':ab,ti OR 'cerebrovascular accidents, acute':ab,ti | 96078 |
|  | #12 OR #13 | 117444 |
|  | #11 AND #14 | 297 |
| **Web of Science** | | |
| 1. | TS=(Lymphocyte) | 196544 |
| 2. | TI=(Lymphoid Cell OR Lymphocyte) | 45647 |
| 3. | AB=(Lymphoid Cell OR Lymphocyte) | 154111 |
| 4. | #1 OR #2 OR #3 | 218744 |
| 5. | TS=(Monocyte) | 93094 |
| 6. | TI=(Monocyte) | 22015 |
| 7. | AB=(Monocyte) | 69010 |
| 8. | #5 OR #6 OR #7 | 93094 |
| 9. | #4 AND #8 | 16237 |
| 10. | TI=(L/M ratio OR lymphocyte to monocyte ratio OR lymphocyte monocyte ratio OR LMR) | 1210 |
| 11. | AB=(L/M ratio OR lymphocyte to monocyte ratio OR lymphocyte monocyte ratio OR LMR) | 7080 |
| 12. | #10 OR #11 | 7419 |
| 13. | #9 OR #12 | 19387 |
| 14. | TS=(Ischemic Stroke) | 107823 |
| 15. | TI=(Ischemic Strokes OR Stroke, Ischemic OR Ischaemic Stroke OR Ischaemic Strokes OR Stroke, Ischaemic OR Acute Ischemic Stroke OR Acute Ischemic Strokes OR Ischemic Stroke, Acute OR Stroke, Acute Ischemic OR Cerebral Stroke OR Cerebral Strokes OR Stroke, Cerebral OR Strokes, Cerebral OR Stroke, Acute OR Acute Stroke OR Acute Strokes OR Strokes, Acute OR Cerebrovascular Accident, Acute OR Acute Cerebrovascular Accident OR Acute Cerebrovascular Accidents OR Cerebrovascular Accidents, Acute) | 53155 |
| 16. | AB=(Ischemic Strokes OR Stroke, Ischemic OR Ischaemic Stroke OR Ischaemic Strokes OR Stroke, Ischaemic OR Acute Ischemic Stroke OR Acute Ischemic Strokes OR Ischemic Stroke, Acute OR Stroke, Acute Ischemic OR Cerebral Stroke OR Cerebral Strokes OR Stroke, Cerebral OR Strokes, Cerebral OR Stroke, Acute OR Acute Stroke OR Acute Strokes OR Strokes, Acute OR Cerebrovascular Accident, Acute OR Acute Cerebrovascular Accident OR Acute Cerebrovascular Accidents OR Cerebrovascular Accidents, Acute) | 102954 |
| 17. | #15 OR #14 OR #16 | 146535 |
| 18. | #13 AND #17 | 242 |

**Table S2: Sensitivity analysis of meta-analysis between LMR and poor functional outcome at 3 months.**

|  | OR | 95%Cl | I² |
| --- | --- | --- | --- |
| Using a fixed-effect model | 0.61 | [0.55, 0.67] | 77% |
| All trials | 0.63 | [0.49, 0.80] | 77% |
| Excluding trials with Guan 2023 | 0.62 | [0.45, 0.86] | 82% |
| Excluding trials with Liu 2017 | 0.67 | [0.51, 0.88] | 68% |
| Excluding trials with Lux 2020 | 0.66 | [0.53, 0.81] | 72% |
| Excluding trials with Pinčáková 2022 | 0.59 | [0.45, 0.78] | 74% |
| Excluding trials with Ren 2017 | 0.61 | [0.46, 0.81] | 81% |
| Excluding trials with Zhang 2024 | 0.59 | [0.46, 0.76] | 78% |

LMR lymphocyte-to-monocyte ration, OR odds ratio, CI confidence intervals

**Table S3: The Newcastle-Ottawa Quality Assessment Scale score for cohort studies.**

| author | Representativeness of the exposed cohort | Selection of the non-exposed cohort | Ascertainment of exposure | Demonstration that outcome of interest was not present at start of study/before ascertainment of exposure | Comparability of cohorts on the basis of the design or analysis | Assessment of outcome | Was follow-up long enough for outcomes to occur | Adequacy of follow up of cohorts | total scores |
| --- | --- | --- | --- | --- | --- | --- | --- | --- | --- |
| Guan  2023 |  |  |  |  |  |  |  |  | 9 |
| Liu  2017 |  |  |  |  |  |  |  |  | 7 |
| Lux  2020 |  |  |  |  |  |  |  |  | 6 |
| Ren  2017 |  |  |  |  |  |  |  |  | 7 |
| Zhang  2024 |  |  |  |  |  |  |  |  | 9 |
| Pinčáková 2022 |  |  |  |  |  |  |  |  | 8 |


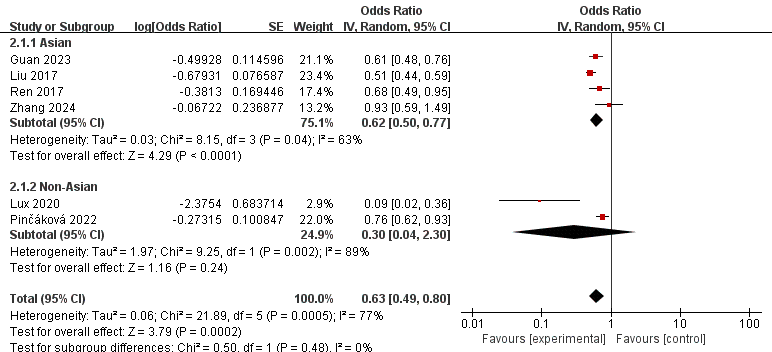


**Figure S1. Forest plot of Asian and non‐Asian populations subgroup analysis.**


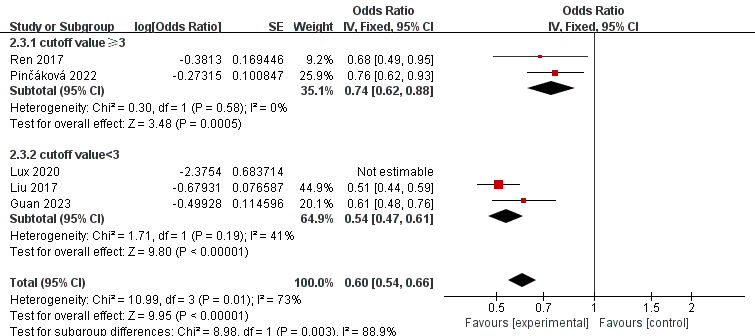


**Figure S2. Forest plot of the best cutoff value subgroup analysis.**


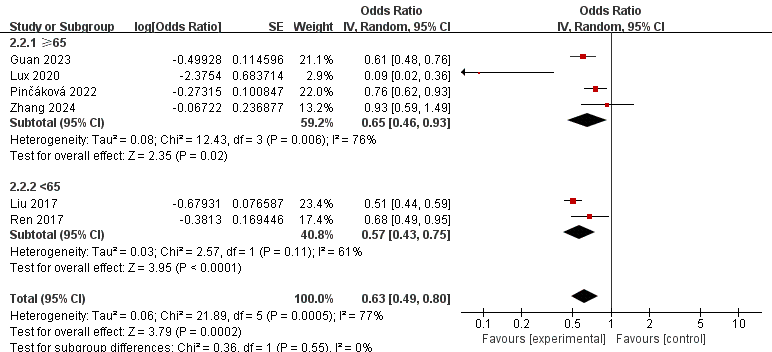


**Figure S3. Forest plot of age subgroup analysis.**


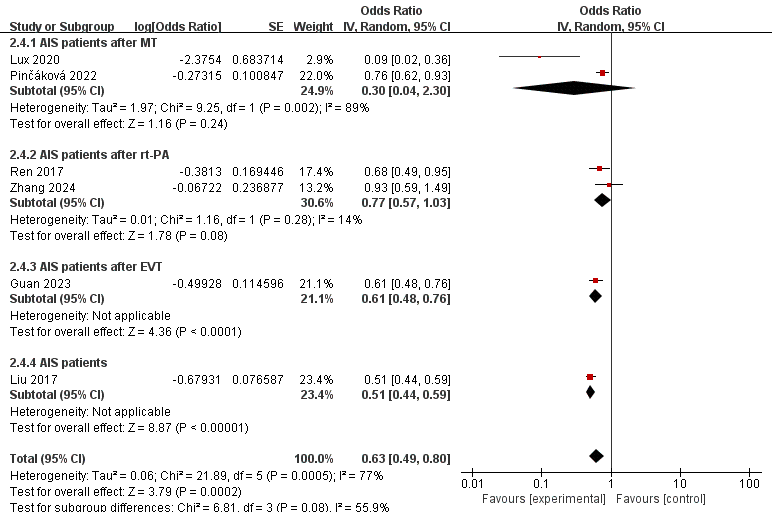


**Figure S4. Forest plot of characteristics of the research object subgroup analysis.**


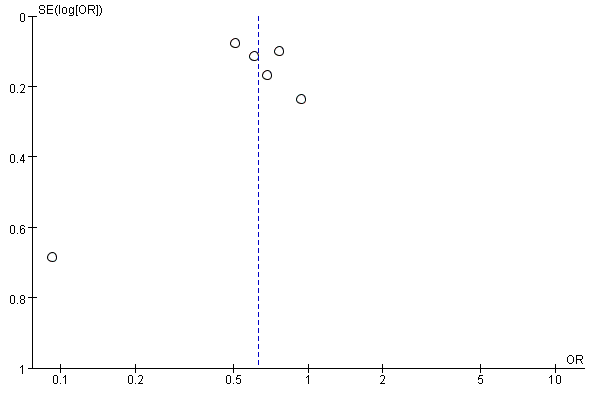


**Figure S5: Funnel plot of the association between Lymphocyte-to-Macrophage ratio and poor functional outcome at 3 months.**


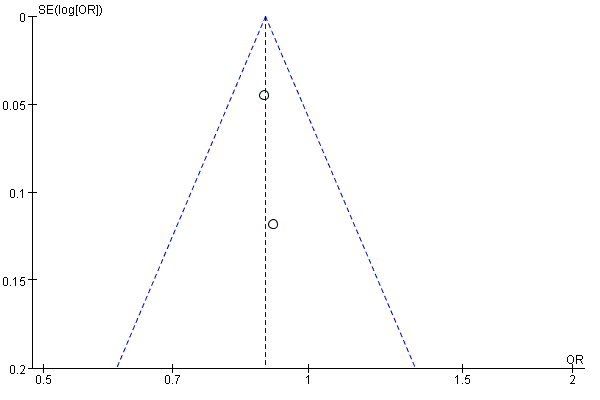


**Figure S6: Funnel plot of the association between Lymphocyte-to-Macrophage ratio and** **moderate to severe stroke.**
